# Commentary: Complement genetic variants and FH desialylation in *S. pneumoniae*-haemolytic uraemic syndrome

**DOI:** 10.3389/fimmu.2025.1707529

**Published:** 2025-11-20

**Authors:** Laura M. Baas, Nicole C. A. J. van de Kar, Marloes A. H. M. Michels, Lambertus P. van den Heuvel

**Affiliations:** 1 Department of Pediatric Nephrology, Radboud University Medical Centre, Amalia Children’s Hospital, Nijmegen, Netherlands; 2 Department of Human Genetics, Radboud University Medical Centre, Nijmegen, Netherlands; 3 Department of Pediatric Nephrology, Emma Children’s Hospital, Amsterdam University Medical Centre, Amsterdam Institute of Infection and Immunity, Amsterdam, Netherlands; 4 Department of Pediatrics/Pediatric Nephrology, University Hospital Leuven, Leuven, Belgium; 5 Department of Development and Regeneration, University Hospital Leuven, Leuven, Belgium

**Keywords:** factor H, Neuraminidase, functional assays, SP-HUS, hemolytic uremic syndrome

## Introduction

Hemolytic uremic syndrome caused by an invasive *Streptococcus pneumoniae* infection (SP-HUS) is a very rare but severe disease of which the pathogenesis is still poorly elucidated. In this recent article, Gómez Delgado and colleagues (1) presented interesting data on the desialylation of members of the Factor H (FH) protein family, which includes FH, Factor H-like 1 (FHL-1), and Complement Factor H Related proteins 1-5 (CFHR1-5), in SP-HUS patients. This desialylation is likely the result of neuraminidases released by *S. pneumoniae* in the circulation of these patients. Furthermore, the authors investigated whether this desialylation of FH affected its key complement-regulatory functions in the complement alternative pathway, to further understand the role of bacterial neuraminidases in the pathogenesis of SP-HUS. As patient samples were limited, they used a biological substitute, which was *in vitro* desialylated FH generated by incubating commercially obtained serum-purified FH with neuraminidase. The authors demonstrated that *in vitro* desialylated FH showed a decreased ability to regulate complement activity on sheep erythrocyte surfaces, while FH’s ability to bind C3b, degrade C3b into iC3b, and its decay accelerating activity remained unchanged. Therefore, the authors hypothesized FH desialylation may have a role in SP-HUS pathogenesis. While we acknowledge the significant contributions this study offers in elucidating the pathogenesis of SP-HUS, we have identified certain technical concerns regarding the execution of the experiments and the interpretation of the data on the desialylation of FH and its functional consequences.

## Discussion

Recently, we assessed the glycosylation status of SP-HUS patient FH using a high-resolution LC-MS/MS-based N-glycoproteomics approach. While we also detected loss of sialylation of FH in our SP-HUS patients, we additionally identified loss of galactose and N-acetylglucosamine residues on the N-glycan structure of FH, which could not have been detected with the lectin blotting method used by Gómez Delgado et al. Because the glycosylation alterations were beyond desialylation and since we had sufficient patient material available, we purified FH from SP-HUS patient sera using immunoprecipitation and evaluated its functional activity (2). We were not able to detect any differences in the functionality of FH derived from SP-HUS patients compared to control FH, as measured by FH’s ability to degrade C3b into iC3b and to regulate complement on the sheep erythrocyte surface. Importantly, we also observed no functional consequence for *in vitro* desialylated FH derived from treating normal human serum with neuraminidase and applying the same FH purification using immunoprecipitation. The resulting *in vitro* desialylated FH preparation specifically lacked sialic acid residues, without detectable loss of galactose and N-acetylglucosamine. We concluded that FH derived from *in vitro* desialylation or SP-HUS patient material, even though having severely affected N-glycans, retains its canonical complement-inhibitory functions. Therefore, we proposed that alterations of FH’s N-glycans, including the loss of galactose and N-acetylglucosamine, are unlikely to directly contribute to complement dysregulation during SP-HUS. Our data, along with other published reports (3–5) consistently demonstrate that the impairment of FH’s N-glycosylation (to varying degrees) does not affect its canonical complement inhibitory activity.

**Figure 1:**
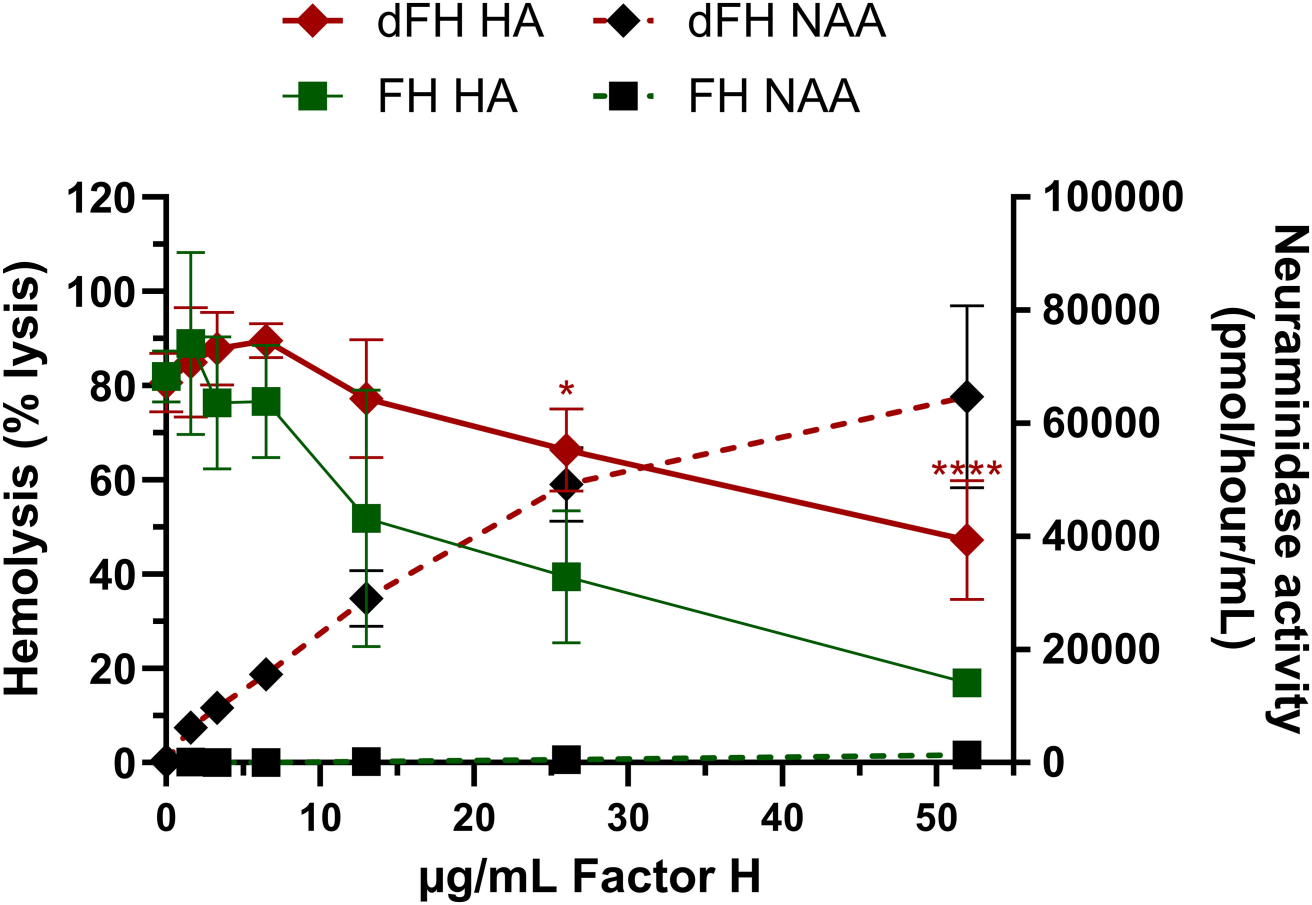
Evaluation of *in vitro* desialylated FH created according to the Gómez Delgado protocol. *In vitro* desialylated FH (dFH) was prepared using the Gómez Delgado protocol (1). In short, commercially available FH (CompTech) was treated with neuraminidase from *C. welchii* for 4 hours at 37°C. Following incubation, the reaction was halted by adjusting the pH to 10. In parallel, FH not treated with neuraminidase (FH) was included as a control. FH functionality was assessed using a sheep erythrocyte hemolytic assay (HA) as described previously (2) to evaluate FH’s capability to protect sheep erythrocytes from complement-mediated lysis. FH and dFH were added in increasing concentrations (0-52 µg/mL). The hemolysis is expressed as % the of the full lysis of erythrocytes in water. Compared to FH (green squares), dFH shows increased hemolysis (red diamonds) suggesting impaired FH complement-regulatory function. Following the HA, the neuraminidase activity assay (NAA) was performed in the supernatants of the HA by fluorometry as described previously (2). dFH shows increased neuraminidase activity in a dose-dependent manner (black diamonds). No neuraminidase activity was detected in the FH sample (black squares). HA data represents the mean ± SD of three independent preparations of dFH/FH measured in two separate experiments. Statistical significance comparing dFH and FH was determined for the HA using multiple unpaired T tests followed by Holm-Sidák’s multiple comparison test; * p ≤ 0.05; and **** p ≤ 0.0001. NAA results are shown as the mean ± SD of three independent experiments. The figure was adapted from previously published data (2), Figures 6A, B.

In contrast, Gómez Delgado and colleagues (1) did report a functional consequence of FH desialylation, as they showed that *in vitro* desialylated FH showed increased lysis of sheep erythrocytes, indicating limited complement-regulatory function. By replicating the method for creating *in vitro* desialylated FH (dFH) reported by Gómez Delgado et al. (1), we indeed noticed increased hemolysis of the erythrocytes with this dFH sample when comparted to FH not treated with neuraminidase (FH) (**Figure 1**) (2). However, we suspected that residual neuraminidase activity in the sample was responsible for this, as the neuraminidases are not removed after the *in vitro* desialylation reaction but are inhibited by a high pH. When we determined the neuraminidase activity in the supernatants of the hemolytic assay using fluorometry, we indeed found residual neuraminidase activity in the dFH samples compared to FH (**Figure 1**) (2).

It is well-established that *C. welchii* neuraminidases have an optimal enzyme activity at pH 5 and are inhibited at pH levels above 9 (6, 7), but the reversibility of this inhibition seems not to have been addressed by the authors. Our data indicates that lowering the pH, as is the case when the desialylated FH sample (pH 10) is diluted in the hemolytic assay buffer (pH 7), restores the neuraminidase activity (at least in part), thus making the neuraminidase inhibition reversible.

In their manuscript, the authors did not present experimental evidence confirming that the enzyme activity was halted following the *in vitro* desialylation reaction and during subsequent functional analyses. The omission of neuraminidases in hemolytic assays is critical, as incomplete inhibition could lead to desialylation of the erythrocyte surfaces, thereby increasing their susceptibility to complement-mediated lysis (8). As a result, the observed increase in erythrocyte lysis may not reflect altered FH functionality due to desialylation of FH itself, but rather reduced recruitment of FH to the erythrocyte surface due to erythrocyte surface desialylation. While the desialylation of cell surfaces may indeed have a role in the disease pathogenesis of SP-HUS, this was outside of the scope of both of our studies and requires a different study design.

In summary, while Gómez Delgado et al. provide observations linking FH desialylation to complement dysregulation and SP-HUS pathogenesis, the lack of controls to exclude residual neuraminidase activity undermines their functional claim. The observed increased hemolysis is likely explained by erythrocyte surface desialylation driven by residual neuraminidase activity, rather than by decreased functionality of the desialylated FH itself. Without conclusive evidence that residual enzyme activity was eliminated, the authors’ interpretation overstates the role of FH desialylation in complement dysregulation in SP-HUS, and their functional data should therefore be viewed with caution.
